# Dendritic cell responses to *Plasmodium falciparum* in a malaria-endemic setting

**DOI:** 10.1186/s12936-020-03533-w

**Published:** 2021-01-06

**Authors:** Triniti C. Turner, Charles Arama, Aissata Ongoiba, Safiatou Doumbo, Didier Doumtabé, Kassoum Kayentao, Jeff Skinner, Shanping Li, Boubacar Traore, Peter D. Crompton, Anton Götz

**Affiliations:** 1 Malaria Infection Biology and Immunity Section, Laboratory of Immunogenetics, National Institute of Allergy and Infectious Diseases, National Institutes of Health, Rockville, MD 20852 USA; 2 Malaria Research and Training Centre, Department of Epidemiology of Parasitic Diseases, International Center of Excellence in Research, University of Sciences, Technique, and Technology of Bamako, 91094 Bamako, Mali

**Keywords:** Dendritic cells, Malaria, *Plasmodium falciparum*, Cytokines, Chemokines, Costimulatory molecules, Myeloid dendritic cells

## Abstract

**Background:**

*Plasmodium falciparum* causes the majority of malaria cases worldwide and children in sub-Saharan Africa are the most vulnerable group affected. Non-sterile clinical immunity that protects from symptoms develops slowly and is relatively short-lived. Moreover, current malaria vaccine candidates fail to induce durable high-level protection in endemic settings, possibly due to the immunomodulatory effects of the malaria parasite itself. Because dendritic cells play a crucial role in initiating immune responses, the aim of this study was to better understand the impact of cumulative malaria exposure as well as concurrent *P. falciparum* infection on dendritic cell phenotype and function.

**Methods:**

In this cross-sectional study, the phenotype and function of dendritic cells freshly isolated from peripheral blood samples of Malian adults with a lifelong history of malaria exposure who were either uninfected (n = 27) or asymptomatically infected with *P. falciparum* (n = 8) was assessed. Additionally, plasma cytokine and chemokine levels were measured in these adults and in Malian children (n = 19) with acute symptomatic malaria.

**Results:**

With the exception of lower plasmacytoid dendritic cell frequencies in asymptomatically infected Malian adults, peripheral blood dendritic cell subset frequencies and HLA-DR surface expression did not differ by infection status. Peripheral blood myeloid dendritic cells of uninfected Malian adults responded to in vitro stimulation with *P. falciparum* blood-stage parasites by up-regulating the costimulatory molecules HLA-DR, CD80, CD86 and CD40 and secreting IL-10, CXCL9 and CXCL10. In contrast, myeloid dendritic cells of asymptomatically infected Malian adults exhibited no significant responses above the uninfected red blood cell control. IL-10 and CXCL9 plasma levels were elevated in both asymptomatic adults and children with acute malaria.

**Conclusions:**

The findings of this study indicate that myeloid dendritic cells of uninfected adults with a lifelong history of malaria exposure are able to up-regulate co-stimulatory molecules and produce cytokines. Whether mDCs of malaria-exposed individuals are efficient antigen-presenting cells capable of mounting an appropriate immune response remains to be determined. The data also highlights IL-10 and CXCL9 as important factors in both asymptomatic and acute malaria and add to the understanding of asymptomatic *P. falciparum* infections in malaria-endemic areas.

## Background

Malaria caused by *Plasmodium falciparum* remains a major public health threat. An estimated 3.4 billion people in 92 countries are at risk of malaria. In 2018, *P. falciparum* caused approximately 228 million malaria cases and 405,000 deaths worldwide, the majority among children in sub-Saharan Africa [1]. The recent scale-up of artemisinin-based combination therapy and insecticide-treated bed nets has reduced the malaria burden in Africa [2]. Worryingly, the trend toward declining malaria cases and deaths observed over the last decade appears to have stalled even before the global COVID-19 pandemic emerged [1]. Now, a recent study suggests that malaria-related deaths in 2020 could increase to more than double those of 2019 if malaria-prevention activities are interrupted due to COVID-19 [3].

Ultimately, a key tool for the control, elimination, and eventual eradication of malaria is an effective vaccine, yet the leading vaccine candidate RTS,S confers only partial, short-lived protection in African children [4]. A consistent finding across several human malaria vaccine trials is a lower immunogenicity in malaria-exposed individuals relative to malaria-naïve individuals [5]. The development of highly effective malaria vaccines has been hindered in part by a poor understanding of the interaction between *P. falciparum* and the human immune system [6], particularly in malaria-endemic areas, where immunoregulatory networks are induced by repeated *P. falciparum* infections [7–12].

In malaria-naïve individuals, *P. falciparum* infections lead to high parasitemia and a strong pro-inflammatory cytokine and chemokine response, referred to as “cytokine storm”, causing fever and other malaria symptoms [13, 14]. However, in endemic areas with repeated exposure, parasitaemia can often be controlled and asymptomatic infections are the rule rather than the exception [15–17]. Although sterile immunity does not appear to be reliably achieved, protective immunity to malaria can be acquired through natural *P. falciparum* infection, but only after years of repeated exposures [18]. Once acquired, this immunity appears to wane rapidly in the absence of ongoing exposure [19].

A crucial part of this process are dendritic cells (DCs). As sentinels of the immune system, DCs are not only important for early cytokine responses but are also essential for bridging and regulating the innate and adaptive immune responses to pathogenic infections and vaccines [20]. DCs reside throughout the body and sample their surroundings for pathogens. Once they phagocytose or encounter pathogenic material they undergo a rapid maturation process and migrate to secondary lymphoid organs to present antigen through MHC molecules to naïve T cells and thus initiate the adaptive immune response [20, 21]. DC maturation is typically characterized by up-regulation of MHC class II (HLA-DR), co-stimulatory markers, and secretion of chemokines and immunomodulatory cytokines such as IL-6, IL-1β, TNF and IL-10. Depending on the nature of their activation, DCs can prime naïve CD4 T cells to become Th1, Th2, or other T helper cell subsets [22] or induce tolerance [23].

The two major human DC subsets, plasmacytoid dendritic cells (pDCs) and myeloid DCs (mDCs), have different roles in initiating and coordinating the immune response. While pDCs secrete high levels of IFNα and TNF upon activation, mDCs up-regulate costimulatory molecules and are highly efficient antigen presenting cells [24].

Despite their essential role in initiating adaptive immune responses to natural infections as well as vaccines, little is known about the role DCs play in the immune response to *Plasmodium*, especially in human malaria infections. Studies both in humans and mouse models have been contradictory [25]. The seminal report on human DCs and *P. falciparum* suggested that DC maturation was blocked by the parasite leading to dysfunction and suboptimal allogenic T cell activation [26]. Conclusions drawn from this report were later challenged by Elliot et al*.* showing the blocking effect was parasite dose-dependent [27]. The experiments in these studies were conducted with monocyte-derived DCs and, therefore, may not be fully representative of human primary DC functions.

Very few studies have addressed DC function using purified primary DCs. One study analysed primary DC functions ex vivo from individuals with acute *P. falciparum* malaria from an endemic area in Papua, Indonesia [28]. The authors analysed DCs within PBMCs (not isolated DCs) and showed that acute infection increased spontaneous apoptosis and dysfunction characterized by decreased costimulatory marker up-regulation, antigen uptake and allogenic T cell activation. Increased spontaneous apoptosis correlated with increased IL-10 levels in the plasma of study subjects with acute malaria. A study investigating DCs from children and adults with asymptomatic malaria from the same cohort did not analyze DC viability but showed unchanged numbers and HLA-DR expression suggesting functional DCs in these asymptomatically infected individuals [29]. Recently, it has been shown that, in malaria-naïve US individuals, enriched primary blood DCs undergo an atypical maturation process characterized by up-regulation of costimulatory molecules and chemokines without significant secretion of cytokines. Despite the lack of inflammatory cytokine secretion, these DCs were capable of inducing a robust Th1-like CD4 T cell response in vitro [30]. During symptomatic infection, this response is likely enhanced by host factors such as reactive oxygen species [31].

These previous studies provide valuable insight into the effect of highly inflammatory conditions during malaria on circulating DCs and early processes during primary DC activation by the parasite in malaria-naïve individuals. However, they do not reveal how isolated DCs from malaria exposed or asymptomatically infected individuals in malaria-endemic areas respond to the parasite. Addressing this question will help us understand whether initial immunological responses to vaccines or natural infections are affected by regulatory networks in individuals living in endemic areas.

Therefore, this study aimed to address whether intense, lifelong malaria exposure or concurrent asymptomatic *P. falciparum-*infections in malaria-endemic Mali have an effect on DC frequency, viability and response to the parasite.

## Methods

### Mali study site, participants and sample collection

Both study sites, Kambila (ClinicalTrials.gov NCT00471302) and Kalifabougou (ClinicalTrials.gov NCT01322581) in Mali, have been described in detail before [32, 33]. Both are rural villages approximately 20 km north (Kambila) and 48 km northwest (Kalifabougou) of Bamako in a region that that typically experiences intense, seasonal *P. falciparum* transmission from July through December each year [32]. For both study sites the enrollment exclusion criteria included anaemia (haemoglobin < 11 g/dL), current use of anti-malarials, corticosteroids or other immunosuppressants, fever > 37.5 °C or evidence of an acute infection, and current pregnancy. Plasma was obtained from US adult donors between the ages of 35 and 70 enrolled in a healthy donor protocol at the NIH Department of Transfusion Medicine, Clinical Center (ClinicalTrials.gov NCT00001846). Eligibility criteria for these donors included no history of malaria in the past 12 months. Further demographic and travel history data were not available from these anonymous donors, but prior *P. falciparum* exposure was unlikely.

The cross-sectional study in Kambila included 35 adults (Table 1), who exhibited no symptoms of malaria and were enrolled between November and December 2016. Asymptomatic *P. falciparum* infections in these individuals were detected by PCR analysis of blood spots at the NIH after in vitro assays had been conducted in Mali. Detailed methods for the detection of *P. falciparum* blood-stage infection by PCR have been described before [33]. Plasma samples from 19 children (Table 1) residing in Kalifabougou were obtained in May 2013 for the healthy baseline time point and during the following transmission season for the acute malaria and 7 days post treatment convalescence time points. Acute malaria was defined as ≥ 2500 asexual parasites/µL, an axillary temperature of ≥ 37.5 °C or self-reported fever within 24 h, and no other cause of fever discernible by physical exam. Acute malaria was treated according to the Malian National Malaria Control Programme guidelines.

**Table 1:** Study cohort

		Subjects	Age	Sex	Symptomatic
		*n*	*Median (IQR)*	*M/F*	
Adults uninfected		27	42 (37–49)	15/12	No
Adults infected		8	51 (46–55)	5/3	No
Children		19	8 (6–9)	11/8	
Healthy baseline					No
Acute infected					Yes
7 days post treatment					No

Peripheral blood was collected by venipuncture into 8 mL sodium citrate-containing cell preparation tubes (BD, Vacutainer CPT Tubes) and transported to the laboratory in Bamako where PBMCs were isolated according to the manufacturer’s instructions. The blood volume was 48 mL for adults and 8 mL for children. PBMCs and dendritic cells were analyzed or isolated immediately. Plasma samples were cryopreserved at -80 °C and later shipped to the NIH. The same sample collection and processing was conducted for the US donors.

### Plasmodium falciparum culture and lysate preparation

Asexual blood stage cultures of the *P. falciparum* strain 3D7 were maintained at 5% haematocrit in RPMI 1640, 25 mM HEPES supplemented with 10 μg/mL gentamicin, 250 μM hypoxanthine, 25 mM sodium bicarbonate, and 0.5% Albumax II under atmospheric conditions of 5% oxygen, 5% carbon dioxide, and 90% nitrogen. Late-stage *P. falciparum*-iRBCs (trophozoites and schizonts) were isolated using MACS Cell Separation LS Columns (Miltenyi Biotec). *Plasmodium falciparum*-iRBCs were washed, resuspended at 1 × 10^6^/µL and lysed by three consecutive freeze/thaw cycles. Uninfected RBC lysates were prepared accordingly and used as negative control. Lysates were stored at − 80 °C until use.

### Dendritic cell enrichment and culture

Myeloid Dendritic Cell Isolation Kits (Miltenyi Biotec) were used to negatively enrich primary peripheral blood mDCs from PBMCs, following the manufacturer’s instructions. Primary mDCs were cultured with RPMI 1640 supplemented with 10% heat-inactivated human AB serum (Valley Biomedicals) at 37 °C and 5% CO_2_. Enriched mDCs were seeded at 1.5 × 10^5^ per well in 96-well tissue culture-treated plates and cultured with *P. falciparum*-iRBC lysates at a ratio of 1:3 (DC/iRBC) for 24 h. Uninfected RBC lysates were used as a negative control. mDCs were then harvested for FACS analysis and supernatants were stored at − 80 °C until shipment to the NIH and cytokine/chemokine analysis.

### Flow cytometry

Flow cytometry was performed using a BD LSR II cytometer (BD Biosciences), and data were analysed with FlowJo 10 (Tree Star). Primary DC subsets were identified using the following labelled monoclonal antibodies: a lineage cocktail (CD3 UCHT1, CD14 HCD14, CD19 HIB19, CD20 2H7 and CD56 HCD56), HLA-DR (L243), CD303 (201A), CD1c (L161), CD141 (M80) and CD16 (3G8). DC apoptosis was quantified by staining a separate PBMC aliquot with monoclonal antibodies against lineage, HLA-DR (L243) and Annexin V as well as 7-AAD. To normalize to microlitres of blood, the total number of PBMCs was determined using a counting chamber after isolation, prior to further analysis and dendritic cell isolation. Assuming this number was close to the number of PBMCs in whole blood, FACS analysis percentages were used to determine cell #/µl of blood. mDC maturation after culture with *P. falciparum*-iRBC lysate was analysed using the following antibodies: HLA-DR (L243), CD80 (2D10), CD86 (IT2.2) and CD40 (5C3). All antibodies, Annexin V and 7-AAD were purchased from BioLegend or BD Biosciences.

### Cytokine and chemokine analysis

To analyse cytokine and chemokine levels in plasma and culture supernatants, the following kits were used: LEGENDplex Human Inflammation Panel 1 (13-plex) (Biolegend), Cytometric Bead Array Human Inflammatory Cytokine Kit (BD Biosciences) and Cytometric Bead Array Human Chemokine Kit (BD Biosciences).

### Statistical analysis

Statistical analyses were performed using Prism 8 (GraphPad Software) and JMP (SAS Institute). Depending on the experimental design, a paired or unpaired t-test with Welch’s correction was performed. For data in Fig. 6 that were mixed paired and unpaired, a linear mixed model ANOVA with Tukey post hoc tests were performed. The statistical tests are described in the figure legends.

## Results

### Study cohort

To address whether lifelong exposure to intense *P. falciparum* transmission or concurrent asymptomatic *P. falciparum*-infections lead to altered frequency or function of DCs, freshly isolated, primary myeloid DC activation by *P. falciparum*-iRBC lysate in vitro was analysed in a malaria-endemic setting in a cross-sectional study. In the rural village of Kambila, Mali, where malaria transmission is intense and seasonal [32], 35 asymptomatic adults were enrolled in the study. Peripheral venous blood was drawn at the peak of the malaria transmission season and transported to a laboratory at the Malaria Research and Training Centre in Bamako, Mali, where in vitro experiments were conducted immediately upon sample arrival. Plasma samples were cryopreserved and shipped to the NIH for cytokine and chemokine analysis. Asymptomatic infection was determined using PCR at the NIH, Rockville. Of the 35 enrolled Malian individuals, 8 were PCR-positive for peripheral *P. falciparum* blood-stage infection (Table 1). A significant difference in age between the two groups was observed: asymptomatically *P. falciparum*-infected adults were on average 9 years older than the uninfected group (uninfected: 42 ± 8; infected: 51 ± 5). Due to naturally acquired clinical immunity to the parasite, adults in this cohort rarely develop symptomatic infections [18]. To characterize cytokine and chemokine plasma levels of symptomatic *P. falciparum*-infections along with asymptomatic infections, plasma samples from 19 children between the ages of 6 and 9 years were added to the study (Table 1). These children resided in a different village than the adult cohort. Malaria endemicity in this village was comparable to the study site of the adult cohort [33]. PBMC subset and in vitro mDC analyses were not performed with samples obtained from children due to the lower blood volumes obtained and lack of access to freshly isolated cells. The groups were mostly gender balanced (Table 1).

### Subset distribution and apoptosis of DCs in malaria-exposed and asymptomatically-infected adults

**Fig. 1:**
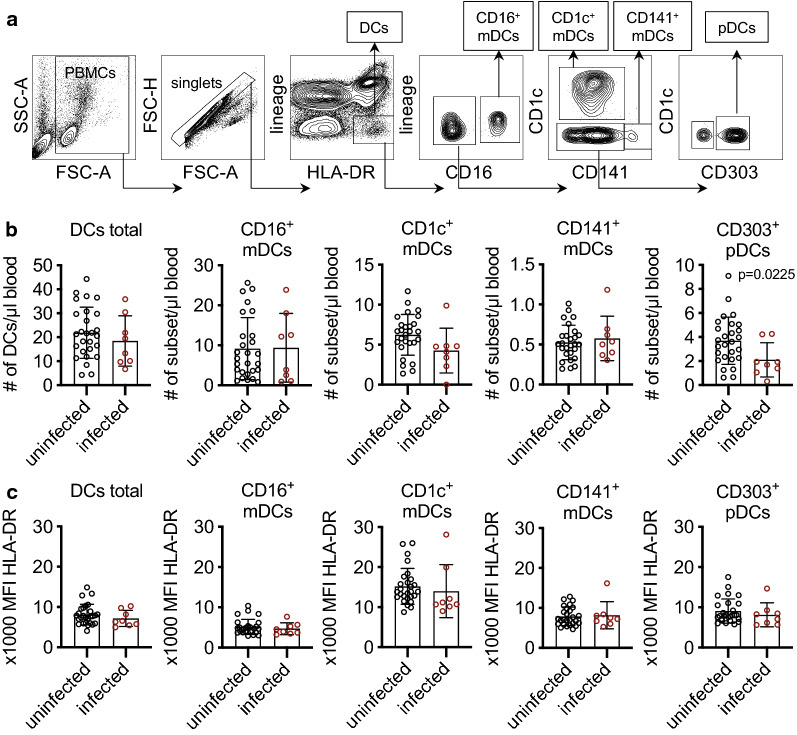
Frequencies and HLA-DR expression of primary blood DC subsets. Cells were gated on viable mononuclear cells (PBMCs) and then further gated on singlets. Blood DCs were identified as Lin^−^ HLA-DR^+^ cells (DCs) and further divided into CD16^+^ and CD16^−^, which were gated on CD1c^+^ and CD141^+^ DCs. CD1c/CD141 double-negative DCs were gated on CD303 to identify pDCs (**a**). Blood DC counts (**b**) and HLA-DR expression as MFI (**c**) were quantified comparing uninfected (n = 27) and asymptomatic *P. falciparum*-infected (n = 8) Malian adults. Each circle represents one individual donor at one time point; bars represent mean with standard deviation. P-value by unpaired t-test. *MFI* median fluorescence intensity

First, enumeration of peripheral blood DC subsets was conducted to identify possible differences in DC numbers between malaria-exposed but uninfected and asymptomatically *P. falciparum*-infected adults. The human peripheral blood DC compartment can be identified by flow cytometry as lineage (CD3, CD14, CD19, CD20 and CD56) negative and HLA-DR positive cells [34]. This population can be further divided into myeloid (mDC) and plasmacytoid DC (pDC) subsets. pDCs can be classified by their expression of CD303. mDCs are composed of two classical subsets, CD1c^+^ and CD141^+^ DCs, and a third subset characterized by the expression of CD16 (Fig. 1a) [35, 36]. This classification was used to assess the frequency of these DC subsets in malaria-exposed but uninfected and asymptomatically *P. falciparum*-infected Malian adults. FACS analysis percentages were used to normalize to subset cell number per µl of blood after determining the total number of PBMCs per sample/volume of blood (Fig. 1). No significant difference in peripheral numbers of total DCs or mDC subsets was observed. pDC numbers in the circulation of asymptomatically *P. falciparum*-infected individuals, however, were found to be on average approximately half of the frequency of pDCs in uninfected individuals (Fig. 1b).

A decrease in HLA-DR surface expression, which might lead to suboptimal T cell priming, has been described in acute malaria patients [28, 37–42]. Therefore, this study aimed to answer whether altered HLA-DR expression was associated with asymptomatic *P. falciparum*-infection. Here, HLA-DR surface levels in uninfected and asymptomatically-infected Malians were found to be comparable (Fig. 1c). As expected, CD1c^+^ mDCs expressed the highest levels of HLA-DR on their surface, while CD16^+^ exhibited low surface HLA-DR (Fig. 1c) [36, 43].

**Fig. 2:**
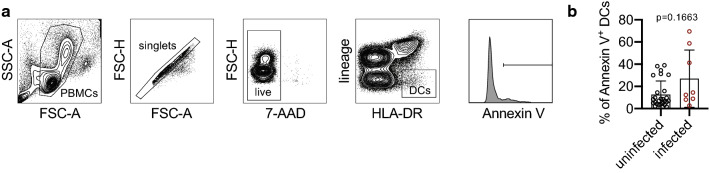
Analysis of primary blood DC apoptosis in uninfected versus asymptomatically infected adults. Cells were gated on viable mononuclear cells (PBMCs), then further gated on singlets and 7-AAD negative (live) cells. Blood DCs were identified as Lin^−^ HLA-DR^+^ cells (DCs) and apoptosis determined by Annexin-V binding assays. Representative dot plots and a histogram are shown (**a**). Percentage of Annexin-V positive DCs in uninfected (n = 27) and asymptomatically infected (n = 8) Malian adults (**b**). Each circle represents one individual donor at one time point; bars represent mean with standard deviation. P-value by unpaired t-test

In patients with acute malaria, it has been reported that peripheral blood DCs undergo apoptosis [28]. In this study, no significant difference of total DC apoptosis was observed between uninfected and asymptomatically infected Malian adults (Fig. 2). The infected group exhibited a rather large variability of DC apoptosis percentages, ranging from 2.75% to 69.5%.

These data suggest that pDC numbers are slightly lower in asymptomatically *P. falciparum*-infected compared to uninfected Malian adults while mDC numbers in circulation remain unchanged.

### mDCs up-regulate costimulatory molecules and secrete IL-10, CXCL9 and CXCL10 in response to P. falciparum in malaria-exposed adults

**Fig. 3:**
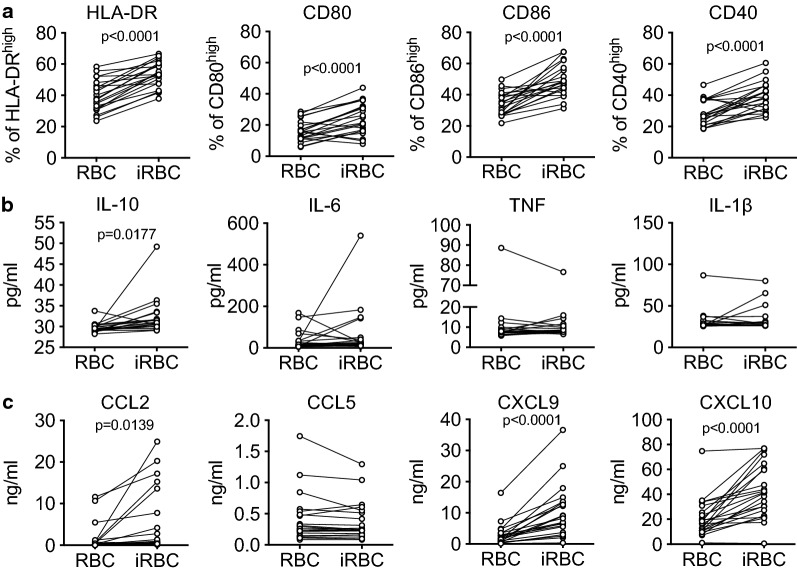
Analysis of *P. falciparum*-induced activation of mDCs from uninfected Malian adults. mDCs were enriched from peripheral blood and incubated with *P. falciparum*-iRBC or uninfected RBC lysate at a ratio of 1:3 [DC:(i)RBC] for 24 h and analysed for surface marker expression (**a**), cytokine (**b**) and chemokine (**c**) secretion (n = 21). P-values by paired t-test

Although it would be informative to analyse pDCs and mDCs from individuals living in an endemic area, we decided to focus only on mDCs for this study to enable a more detailed assessment and their ability to efficiently prime naïve T cells. A limitation of commonly conducted activation analyses using whole PBMCs are the various, possibly regulatory, cell types present in PBMCs. To avoid uncontrollable effects of other cell types, mDCs were enriched by negative selection depleting cells bound to magnetic beads with lineage-specific antibodies. Enriched mDCs were comprised of CD1c^+^ and CD141^+^ mDC subsets. Enrichment and in vitro incubation with *P. falciparum* blood-stage lysates were undertaken with freshly isolated cells to minimize the effects of handling, freezing, and thawing the cells. DC surface marker expression as well as cytokine and chemokine secretion commonly associated with mDC activation were analysed after stimulation with *P. falciparum*-iRBC lysate for 24 h. Uninfected RBC lysate was used as a negative control. To minimize batch effects mDC responses to the parasite were assessed for all 35 enrolled uninfected and asymptomatically *P. falciparum*-infected adults simultaneously. However, due to generally low numbers of DCs in circulation, some samples had too few DCs to successfully execute the assay. Out of the 27 uninfected adults, 21 had sufficient mDC numbers to conduct the experiments (Fig. 3).

mDCs isolated from uninfected Malian adults were able to up-regulate the activation markers HLA-DR, CD80, CD86 and CD40 upon stimulation with parasite lysate (Fig. 3a). Malian mDCs did not secrete significant amounts of the inflammatory cytokines IL-1β, IL-6 or TNF, but a slight but significant increase of IL-10 was measured in the culture supernatant when mDCs were stimulated with *P. falciparum*-iRBC lysate (Fig. 3b). mDCs from malaria-exposed adults also secreted the Th1-associated chemokines CXCL9 (MIG) and CXCL10 (IP-10) as well as CCL2 but no CCL5 (Fig. 3c).

**Fig. 4:**
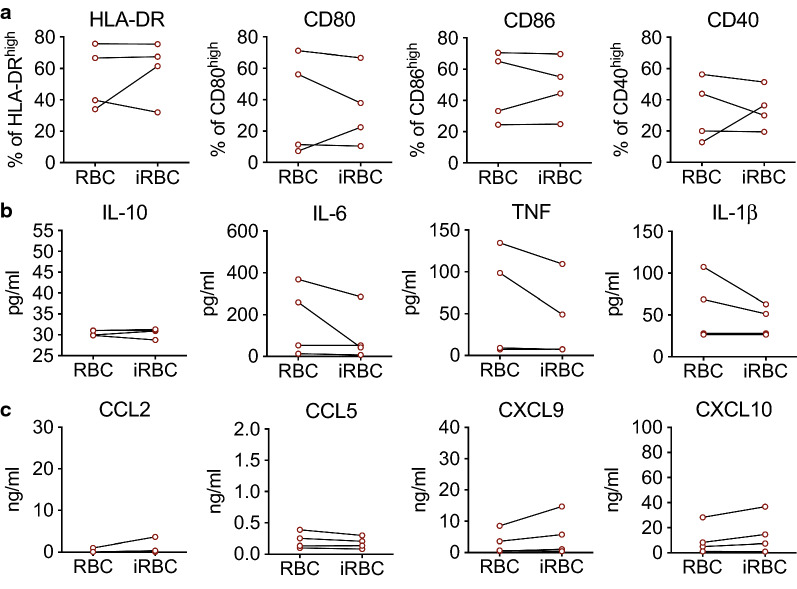
Analysis of *P. falciparum*-induced activation of mDCs from asymptomatically infected Malian adults. mDCs were enriched from peripheral blood and incubated with P. falciparum-iRBC or uninfected RBC lysate at a ratio of 1:3 [DC:(i)RBC] for 24 h and analysed for surface marker expression (**a**), cytokine (**b**) and chemokine (**c**) secretion. All non-significant by paired t-test

Of the 8 asymptomatically infected individuals, only 4 had sufficient mDC numbers for analysis. Among these 4 individuals, no trends or significant differences were detected (Fig. 4).

### Plasma cytokine and chemokine levels in asymtomatically-infected adults and children with acute malaria

Next, plasma cytokine and chemokine levels in the described cohort of asymptomatically *P. falciparum*-infected and uninfected Malian adults were analysed by bead-based immunoassays. In addition to the cytokines and chemokines tested in DC supernatants, cytokines commonly associated with T cell responses were tested.

**Fig. 5:**
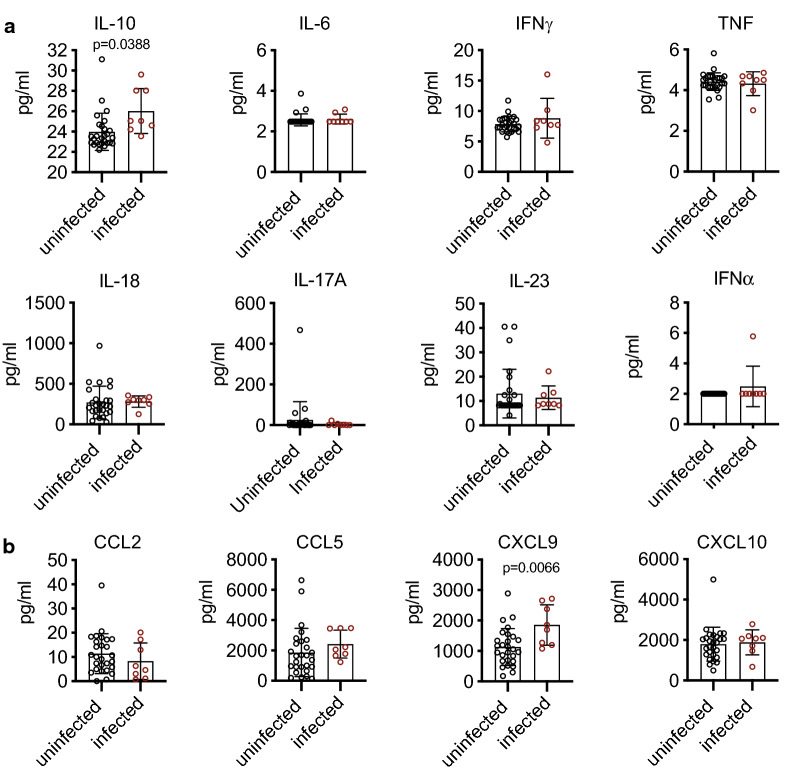
Plasma cytokine and chemokine analysis in uninfected and asymptomatically P. falciparum-infected adults. Cytokine (**a**) and chemokine (**b**) concentrations in plasma were analyzed in asymptomatic uninfected (n = 27) or *P. falciparum*-infected Malian adults (n = 8). Each circle represents one individual donor at one time point; bars represent mean with standard deviation. P-values by unpaired t-test

Slightly elevated levels of IL-10 (Fig. 5a) and CXCL9 (Fig. 5b) were detected in plasma of asymptomatically infected compared to uninfected adults. This observation corroborates our finding that DCs in these individuals respond to the parasite with IL-10 and CXCL9 in vitro (Fig. 4). All other analysed cytokines and chemokines, namely IL-6, TNF, IL-1β, IL-18, IFNγ, IFNα, CCL2, CCL5 and CXCL10, were comparable between the two groups (Fig. 5), suggesting they were at baseline levels in both groups.

**Fig. 6:**
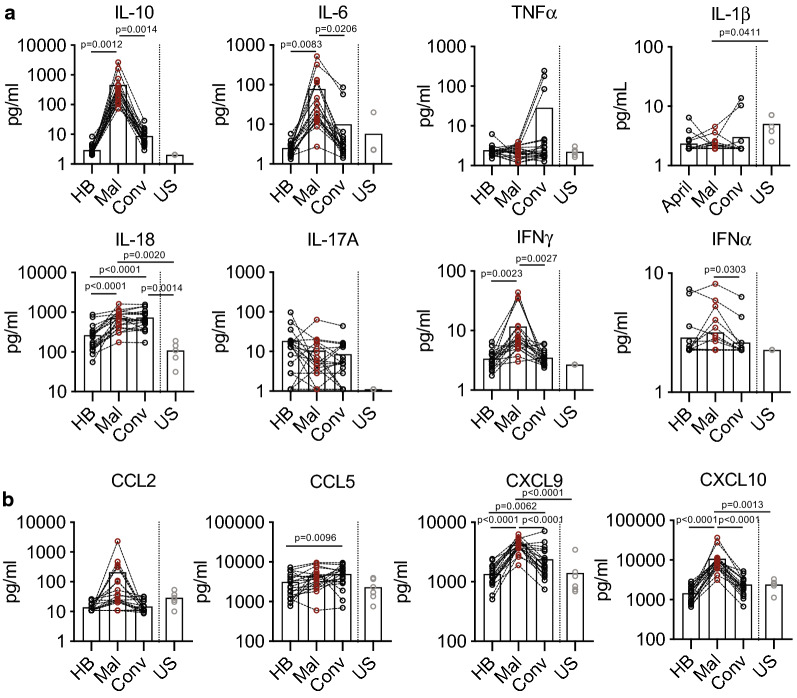
Plasma cytokine and chemokine analysis before, during and after symptomatic *P. falciparum* infection in children. Cytokine (**a**) and chemokine (**b**) concentrations in plasma were analysed at healthy baseline (HB), during symptomatic acute infection (Mal) and 7 days post treatment (Conv) in Malian children (n = 19) and US adults (n = 5–6). Each circle represents one individual donor and time point; bars represent mean. P-values by linear mixed model ANOVA

Finally, to compare these responses to symptomatic malaria, plasma cytokine and chemokine levels were measured longitudinally in Malian children before (healthy baseline, HB), during (malaria, Mal) and after (convalescence, Conv) an acute symptomatic malaria episode. Plasma obtained from US adults was used as a control. As expected, most inflammatory cytokine and chemokine levels tested increased significantly during acute malaria and decreased to baseline levels at the convalescent time point 7 days post treatment. Similar to asymptomatically infected adults (Fig. 5a), plasma IL-10 levels exhibited the largest increase with a 150-fold increase during acute malaria compared to baseline (Fig. 6a). Mean IL-6 and IFNγ levels increased roughly 30- and 3.5-fold, respectively. Surprisingly, the inflammatory cytokines IL-1β and TNF did not show significantly higher plasma levels during acute malaria. Overall IFNα levels in plasma were low, but a significantly higher amount of this cytokine was measured during acute malaria compared to convalescence (Fig. 6a). The Th1-associated chemokines CXCL9 and CXCL10 exhibited a robust increase in plasma levels during acute malaria (Fig. 6b).

Most plasma cytokine and chemokine levels returned to baseline by the convalescent timepoint, with the exception of IL-18, CCL5 and CXCL9 (Fig. 6). Taken together, these data indicate that IL-10 plays a prominent role in the cytokine response to *P. falciparum* in malaria-endemic settings.

## Discussion

The biology of primary human DCs is understudied due in part to their low frequencies in peripheral blood and the technical difficulties of relevant experimental assays. The understanding of primary human DC responses to *P. falciparum* in particular remains very limited (reviewed in [44]). In light of evidence that the leading malaria vaccine candidate RTS,S confers only partial, short-lived protection in African children [4], it is of great importance to gain a better understanding of DC responses, particularly in malaria-endemic settings. This study attempted to address this knowledge gap by analysing DC frequency, viability and responsiveness to *P. falciparum* in a malaria-endemic setting.

The findings suggest that mDC numbers in circulation remain stable during asymptomatic malaria in Malian adults. pDC frequencies, however, were lower in individuals with asymptomatic *P. falciparum* infections compared to uninfected individuals. Several other studies have reported lower numbers of circulating DCs in acute malaria patients in both children and adults [28, 37, 45–47]. In asymptomatic *P. falciparum* infections in children and adults in Papua, Indonesia, DC numbers were found to be unchanged, including pDC frequencies [29]. This discrepancy could be explained by the distinct study sites. Malaria generally presents differently in Asia and in West Africa, likely due to a number of differences including transmission intensity and availability of resources [48, 49]. Individuals with asymptomatic infections in our cohort were significantly older than the uninfected controls. Since pDC, but not mDC, numbers in circulation decline with age [50–52], lower pDC frequencies in this group could be attributed to age rather than asymptomatic malaria. It is also unclear whether decreased numbers of DCs in circulation is due to DC apoptosis [28], or migration of activated DCs out of circulation to target tissues. In this study, a higher percentage of apoptotic total DCs in asymptomatically infected compared to uninfected adults was not observed. Since the percentage of apoptotic pDCs was not assessed, however, the total DC data could mask a difference between groups in pDC apoptosis. The limited number of infected individuals and rather large variability of DC apoptosis limits the interpretability of these data. Additional experiments will be needed to address whether peripheral blood pDCs undergo apoptosis in asymptomatically infected individuals.

A few studies have reported an increase in CD141^+^ mDCs in children with acute malaria [38, 39, 53]. This increase was shown to be induced by Flt3 ligand, which was in turn induced by a signaling cascade staring with type I interferon. Confirming previous findings in asymptomatic adults with malaria [29], no such increase was observed in the present study, suggesting that a threshold inflammatory response that includes type I interferon may be necessary to trigger expansion of CD141^+^ mDCs through Flt3 ligand.

As observed previously with a subset of the data [30], DCs isolated from uninfected Malian adults were able to up-regulate the activation markers HLA-DR and CD86 upon stimulation with parasite lysate. Likely due to the larger sample size, here CD80 and CD40 were also found to be significantly up-regulated. Similar to responses seen with DCs from malaria-naïve US donors [30], mDCs obtained from Malians did not secrete significant amounts of the inflammatory cytokines IL-1β, IL-6 or TNF. Unlike the cytokine responses exhibited by DCs from US donors however, a slight but significant increase in IL-10 was measured when DCs from Malian individuals were stimulated with *P. falciparum*-iRBC lysate. Whether mDCs from malaria-naïve US individuals are also capable of responding to the parasite with IL-10 secretion will have to be addressed in future experiments. The absence of a significant increase in the previous study could be due to a lower sample size and does not permit further conclusions at this time. Comparable to the response exhibited by DCs from US donors to the parasite, DCs from Malians secreted the Th1-associated chemokines CXCL9 and CXCL10 as well as CCL2 but no CCL5. Except for IL-10 secretion, in vitro DC responses to the parasite in malaria-exposed uninfected Malians seem, at least qualitatively, comparable to malaria-naïve US DCs. Future studies will have to address whether up-regulation of costimulatory molecules and chemokines in these DCs translates into effective T cell priming in malaria-endemic areas.

A number of studies have assessed cytokines in the plasma of acute malaria patients, concluding that the so called “cytokine storm”, characteristic for *P. falciparum* infections, contributes to pathology [54]. Here, an increase of most inflammatory cytokines tested during a first febrile malaria episode of the season in Malian children compared to their baseline and 7 days post treatment (convalescence) was also found. The data corroborates previous observations for IL-18, IFNγ and IL-10 in the same cohort and adds IL-6 and IFNα as being up-regulated while IL-1β, IL-17A and TNF remained unchanged [55]. TNF levels were previously shown to be elevated during malaria in this cohort. This discrepancy is surprising and might be due to a difference in age, the present study assessed children between the ages of 6 and 9 while the previous study focused on children between the ages of 3 to 12. Another difference was the year of the blood draw, 2011 vs 2013, and kit used to analyse cytokine levels in plasma. This highlights the importance of repeated assessment of parameters at different time points to be able to draw accurate conclusions.

Plasma chemokine levels during human malaria are less well documented. Similar to other studies in Africa [56, 57], this study reports high levels of CXCL9 and CXCL10 in the plasma of children with acute malaria and significantly higher CXCL9 levels in asymptomatically-infected compared to uninfected adults. CXCL10 has been shown to have a negative effect on the development of immunity and promotes severe outcomes in murine [58, 59] and human [60] malaria. Similar to this study’s findings with mDCs stimulated with *P. falciparum*-iRBC lysates*,* CXCL9 and CXCL10 have been reported to exhibit increased expression in murine splenic DCs during *Plasmodium chabaudi* infection, with no significant increase in inflammatory cytokine gene expression [61]. Comparable to the findings in this study, mDCs from malaria-naïve individuals secrete high levels of CXCL9 and CXCL10 in response to the parasite in vitro. Interestingly, these chemokines are induced to a greater extend by the parasite than by LPS, the gold standard DC activator [30], suggesting that these chemokines may be characteristic of *P. falciparum* infections. Similar to the “cytokine storm”, inflammatory chemokines like CXCL9 and CXCL10 are likely important to mount an effective immune response to the parasite early during the infection, but may be detrimental under certain circumstances.

In addition to CXCL9 and CXCL10, we observed that low levels of IL-10 were secreted by mDCs in Malian adults upon stimulation with the parasite. That IL-10 was detectable in the plasma of asymptomatically infected adults and at high levels in children with acute malaria is consistent with an important role for this regulatory cytokine in malaria. Indeed, Il-10 has been extensively described in murine malaria models and human studies as an important regulatory factor (reviewed in [62]). Although commonly believed to be produced mainly by Tr1 cells, our data suggests that mDCs may be a significant contributor to the overall IL-10 response during *Plasmodium* infection.

Studies that assess plasma cytokine or chemokine levels in asymptomatic malaria infections are scarce. In this study, IL-10 and CXCL9 were significantly elevated in asymptomatic *P. falciparum*-infected Malian adults. While children with asymptomatic malaria have increased IFNγ, TNF and IL-4 levels [63], IL-10 and CXCL10 were elevated in asymptomatically infected pregnant women [64–66]. This study’s data suggest that asymptomatically infected adults in endemic areas exhibit elevated levels of IL-10 as well. CXCL9 was not assessed by these studies and, together with IL-10, might represent a potential biomarker to detect asymptomatic infections.

Limitations of this study include the low number of infected individuals enrolled, the cross-sectional design and the lack of data on DC-T cell interactions. Future studies should include a larger sample size, and longitudinal analysis of DC responses and functionality that include T cell activation. The Malian study site is particularly well suited for such studies due to the sharply demarcated and predictable malaria transmission season which enables the analysis of DCs from uninfected individuals before the malaria season and the same individuals as they become infected during the ensuing malaria season. Despite these limitations, the present study provides valuable insight into baselines DC frequencies and responses as well as in vivo cytokine and chemokine responses to the parasite in a malaria-endemic setting.

## Conclusions

Although far from complete, together with the report on peripheral blood DC frequencies in asymptomatic malaria in Papua, Indonesia [29], this study begins to address if mDC responses are altered by malaria-exposure. The data suggest, that mDCs from adults living in malaria-endemic Mali are able to respond to *P. falciparum*-iRBCs. However, whether these responses translate into efficient T cell activation remains to be determined. This report also suggests that IL-10 and CXCL9 are upregulated during asymptomatic malaria, which may be of interest given the high prevalence of asymptomatic infection in endemic areas [67, 68].

## Data Availability

The datasets used and/or analysed during the current study are available from the corresponding author on reasonable request.

## Abbreviations

DC: Dendritic cell
iRBC: Infected red blood cell
mDC: Myeloid dendritic cell
pDC: Plasmacytoid dendritic cell
RBC: Red blood cell
